# Cryptogenic stroke and patent foramen ovale: endeavoring for clarity

**DOI:** 10.3389/fneur.2024.1533232

**Published:** 2025-01-14

**Authors:** Tohid Amini

**Affiliations:** ^1^School of International Medicine, Istanbul Medipol University, Istanbul, Türkiye; ^2^Medipol University Hospital, Istanbul, Türkiye

**Keywords:** cryptogenic stroke (CS), PFO (patent foramen ovale), PFO closure, stroke recurrence, biomarkers, perioperative stroke, RoPE score and PASCAL classification, PFO detection

## Abstract

This review aims to summarize current knowledge and highlight recent findings on the association between cryptogenic stroke (CS) and patent foramen ovale (PFO). By presenting sometimes conflicting data, the review underscores the necessity for further research to clarify the complex mechanisms behind PFO-related CS and optimize its management. Results from research identifies specific conditions and scores, such as the risk of paradoxical embolism (RoPE) score, that help assess the likelihood of PFO-related cryptogenic stroke and guide treatment decisions. PFO closure has demonstrated substantial benefits in select cases, especially those with high-risk PFO features, though complications such as atrial fibrillation were frequently documented. Biomarker measurements, such as reduced total homocysteine (tHcy) level after PFO closure or high D-dimer levels indicating a higher risk of stroke recurrence, represent newer areas of study with a promising future in medical practice. Cryptogenic stroke (CS) remains a diagnostic challenge. This article reviews the current understanding of PFO-related CS, focusing on the interplay of concomitant pathological conditions, PFO closure, stroke recurrence, and some of the related biomarkers.

## Introduction

The TOAST trial classifies ischemic strokes into five subtypes, (1) large-artery atherosclerosis, (2) cardioembolism, (3) small-vessel occlusion, (4) stroke of other determined etiology, and (5) stroke of undetermined etiology also known as cryptogenic stroke (CS) (1).

The incidence of CS associated with patent foramen ovale (PFO) varies across studies. Some reports suggest PFO is found in approximately 40–50% of CS cases, while in the general population, its prevalence is about 20–25% (2). However, PFO can also be incidental and present in other pathological conditions, including migraine, stroke or transient ischemic attack (TIA), hypoxia-induced events, obstructive sleep apnea, high-altitude pulmonary edema, and platypnea-orthodeoxia syndrome (3). Notably, the older the individual, the larger the right-to-left shunt (3). PFO can also allow venous gas bubbles to enter the arterial system, leading to arterial gas embolism (AGE), a clinical feature of decompression illness (4).

The enigmatic nature of cryptogenic stroke highlights the challenges physicians face in identifying its primary causes. With high prevalence of PFO in CS, understanding the risks associated with PFO is crucial to improving insights into CS and its underlying pathological mechanisms.

## Patent foramen ovale and cryptogenic stroke

In embryonic development, the pulmonary circulation is bypassed since gas exchange does not occur in the lungs. During this stage, the right atrium, which has higher pressure, shunts blood to the left atrium, where pressure is lower, through the foramen ovale. However, after birth, the closure of this shunt is critical. Failure of the foramen ovale to close properly leads to a condition known as patent foramen ovale (PFO) (5).

PFO is estimated to exist in approximately 25% of the general population (6). Clinical trials suggest that PFO is present in about 50% of cryptogenic stroke cases which makes it one of the most common etiologies associated with CS or embolic stroke of undetermined source (ESUS) (7).

In the SAFAS study, which examined the prevalence of PFO in strokes, 10% (23 out of 229 cases) were found to be PFO-linked. However, the study suggests that this finding may be due to its focus on patients with large PFOs. The study also highlighted that PFO-associated strokes occur at younger ages compared to non-PFO strokes (58 vs. 69 years, *p* < 0.001). Additionally, the left atrial volume index (LAVI) was lower in PFO-linked cases (25 vs. 32, *p* = 0.023) (8). Another study by Park et al. (9) found that age was not a significant factor in differentiating PFO-positive and PFO-negative CS cases (56.0 vs. 53.6 years; *p* = 0.087).

### Scoring system

High risk PFOs are more likely to be causative factors in cryptogenic strokes (10). In the DEFENSE-PFO trial, a high-risk PFO was characterized as either a defect larger than 3 mm or a PFO associated with atrial septal aneurysm (ASA) demonstrating hypermobility of the septum during the Valsalva maneuver, leading to a significant increase in PFO size (11).

The risk of paradoxical embolism (RoPE) score and the PASCAL classification are key tools in evaluating the relationship between patent foramen ovale (PFO) and cryptogenic stroke (CS). The RoPE score predicts the probability that a PFO is responsible for a cryptogenic stroke by assessing clinical and imaging factors. These factors include patient age, history of stroke risk factors, and imaging findings. A higher RoPE score suggests a stronger association between the PFO and stroke which aids in determining whether the PFO might be a causal factor (Table 1) (12).

**Table 1:** RoPE score components by Kent et al. (12).

RoPE score components	
No history of hypertension	+1
No history of diabetes	+1
No history of stroke/TIA	+1
Nonsmoker	+1
Cortical infarct, on imaging	+1
Age	
18–29	+5
30–39	+4
40–49	+3
50–59	+2
60–69	+1
≥70	0

On the other hand, the PASCAL classification evaluates the anatomical and physiological characteristics of the PFO, such as the size of the defect and the shunt type. This classification is particularly useful for assessing the embolic potential of a PFO and identifying patients at higher risk of adverse events. Being used together, these tools provide a framework and a general idea for diagnosis and management decisions regarding PFO-related cryptogenic strokes (Table 2) (12). The case report by Patel et al. (13) was analyzed using the RoPE score and PASCAL classification.

**Table 2:** PASCAL classification system by Kent et al. (12).

High RoPE score (≥7)	High risk PFO feature (LS and/or ASA)	PFO-related stroke
Absent	Absent	Unlikely
Absent	Present	Possible
Present	Absent	Possible
Present	Present	Probable

RoPE, risk of paradoxical embolism; LS, large shunt; ASA, atrial septal aneurysm; PASCAL, PFO-associated stroke causal likelihood; PFO, patent foramen ovale.

For the 14-year-old male patient who was presented with cryptogenic stroke:

1. Age: The patient is 14 years old, which falls into the RoPE score age bracket of 18–29 years, earning the maximum 5 points.
2. Absence of hypertension: No history of hypertension (+1 point).
3. Absence of diabetes: No history of diabetes (+1 point).
4. No history of stroke/TIA: The patient had no prior neurological events (+1 point).
5. Non-smoker: The patient is a nonsmoker (+1 point).
6. Cortical infarct on imaging: Neuroimaging revealed an acute ischemic infarct in the middle cerebral artery (MCA) territory, which qualifies as a cortical infarct (+1 point).

#### Total RoPE score: 10/10

A RoPE score of 10 strongly suggests that the PFO is pathogenic rather than incidental (estimated probability of 88–92% that the PFO is related to the stroke).

#### PASCAL classification

The PASCAL classification incorporates the RoPE score and evaluates the anatomical and physiological characteristics of the PFO:

1. High RoPE score (≥7): This patient scores 10 on the RoPE scale which fulfills this criteria.
2. High-risk PFO Features: TEE revealed a marked right-to-left shunt through the PFO. This anatomical characteristic is classified as high-risk.

#### PASCAL classification: “probable” PFO-related stroke

The PASCAL classification indicates a strong likelihood that the PFO contributed to the stroke.

### PFO detection techniques

**Figure 1:**
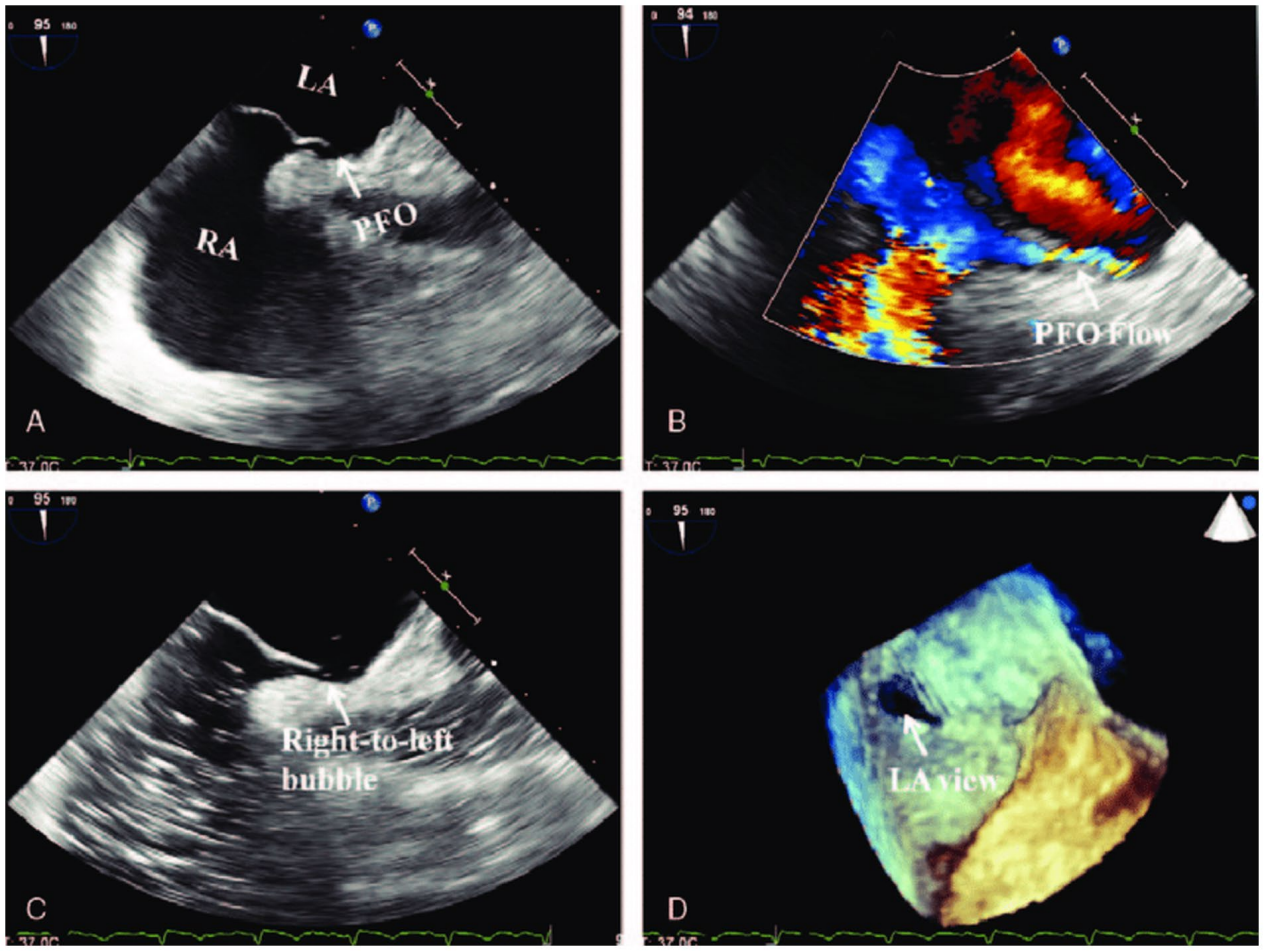
“Transesophageal echocardiography (TEE) confirming the PFO (arrow) (43). **(A)** The two-dimensional TEE in the biatrial view detected a separation between the primum and secundum atrial septum. **(B)** Color Doppler demonstrated bilateral but mainly right-to-left flow by decreasing the color gain and wall filtration. **(C)** Contrast TEE revealed right-to-left shunt after the contrast agent (6 mL of 1% injection vitamin B6 and equal volume of 5% sodium bicarbonate solution) was administered through the dorsal vein of right hand. **(D)** Real-time three-dimensional TEE confirmed the PFO. LA, left atrium; PFO, patent foramen ovale; RA, right atrium” by (Source Publication) is licensed under the Creative Commons Attribution-ShareAlike 4.0 International License.

Transesophageal echocardiography (TEE) is considered the gold standard for the diagnosis of PFO due to its superior accuracy and versatility (Figure 1) (14). However, performing the Valsalva maneuver during TEE can be technically challenging because of the sedative effects used during the procedure. A study by Yamashita et al. (15) demonstrated that inferior vena cava (IVC) compression is an effective and non-inferior alternative to the conventional Valsalva maneuver for PFO detection (*p* < 0.05). This technique avoids the challenges faced after sedation and has improved diagnostic accuracy. Additionally, injecting contrast medium through femoral veins has been shown to significantly enhance detection rates compared to antecubital injections (16). The number of injections also positively correlates with TEE sensitivity, providing a reliable approach for increasing detection (17).

Transcranial Doppler ultrasound, is another technique with the advantage of being non-invasive. Although it does not provide the same anatomical detail as TEE, its non-invasive method, makes it a good choice for initial screening and for patients where TEE is contraindicated (17).

### Paradoxical embolism

Paradoxical embolism is a significant complication associated with PFO. The presence of a right-to-left shunt through the PFO increases the risk of embolic events by allowing thrombi to bypass the lungs and enter the systemic circulation (18). A systematic review reported that the overall 30-day incidence of adverse events following an impending paradoxical embolism (IPDE) was approximately 18% (19).

It is worth mentioning that Catastrophic antiphospholipid syndrome (APS), which is a sever form of antiphospholipid syndrome (APS) resulting in multiple blood clots, can predispose to paradoxical embolism with a concomitant PFO (20).

### Concomitant pathological conditions

Underlying thromboembolic conditions can exacerbate the risk of embolic strokes, especially in the presence of a PFO. The interaction between PFO and hypercoagulability in stroke patients was explored in a study that found no significant association between them (21). The prevalence of hypercoagulability was similar among stroke patients with and without PFO (21).

May–Thurner syndrome (MTS) is characterized by the compression of the iliac vein by an overlying artery, typically in the pelvic region. This venous compression can lead to deep vein thrombosis (DVT) and, in combination with a PFO, increases the risk of ischemic stroke (22). Compression of left common iliac vein by the right common iliac artery is typical of this syndrome (23).

Additionally, the co-occurrence of atrial fibrillation (AF) and PFO has been frequently documented. A right-to-left shunt in patients with PFO and AF significantly raises ischemic stroke risk, while a left-to-right shunt is associated with reduced stroke risk (24).

### Stroke recurrence

Stroke recurrence is a major concern in ischemic stroke management, both in the short and long term. A cohort study from Haukeland University Hospital reported a total recurrence rate of 14.2% over 9 years, associated with higher mortality (HR = 2.25, 95% CI = 2.04–3.18) (25).

A meta-analysis found no association between PFO and increased risk of recurrent stroke or transient ischemic attack (TIA), with a risk ratio of 1.18 (95% CI = 0.78–1.79, *p* = 0.43) for the combined outcome of recurrent stroke/TIA and a risk ratio of 0.85 (95% CI = 0.59–1.22, *p* = 0.37) for recurrent strokes (26).

D-dimer levels, which indicate clot breakdown, are crucial for assessing stroke risk. Elevated D-dimer levels (>1.0 mg/L) are associated with significantly higher rates of recurrent ischemic events following cryptogenic stroke. This was especially pronounced in patients with PFO, with an adjusted hazard ratio (aHR) of over 4.0 (95% CI = 1.63–10.2) compared to D-dimer levels <0.5 mg/L. In contrast, PFO-negative patients with D-dimer >1.0 mg/L had a lower aHR of 1.34 (95% CI = 0.63–2.86) (27). Elevated D-dimer levels were found to increase the risk of all-cause mortality in patients without PFO. Additionally, patients with high D-dimer levels exhibited a heightened risk of pulmonary thromboembolism, regardless of the presence of PFO (28).

Regarding the stroke recurrence in young patients (aged 18–45 years), a study by Arauz et al. (29) showed no evidence of escalated risk for stroke recurrence in presence of PFO among young patients.

### Perioperative stroke

Strokes occurring during surgery or within 30 days postoperatively are classified as perioperative strokes (30). An analysis of the National Readmission Database (NRD) found an increased risk of perioperative stroke and mortality in patients with atrial septal defect (ASD) or PFO. For example, skin and burn surgeries showed a 30-day stroke rate of 0.80% in ASD/PFO patients compared to 0.02% in non-ASD/PFO patients, with an adjusted odds ratio (aOR) of 27.94 (*p* = 0.001) (31).

A meta-analysis by Hobbes et al. (32) supported the increased perioperative stroke risk associated with PFO but found no evidence that PFO directly increased long-term adverse outcomes in perioperative strokes.

## Patent foramen ovale closure

PFO closure has been shown to reduce the recurrence of strokes in appropriately selected patients. High-risk PFO is a primary factor for recommending percutaneous closure. In patients aged ≤60 years, percutaneous closure may be indicated in cases of paradoxical embolism or a history of antithrombotic therapy. For patients older than 60 years, a history of thromboembolic disease is an important consideration in deciding whether to proceed with percutaneous closure (33).

- In a study, 143 patients (29.3%) aged ≤60 years underwent PFO closure. The key indications included detection of high-risk PFO, criteria for paradoxical embolism, and prior use of antithrombotics.
- In the >60 years group, 24 patients (19%) underwent PFO closure, with indications including a history of pulmonary thromboembolism, predisposition to thromboembolic disease, criteria for paradoxical embolism, and high-risk PFO.
- The study also noted a low recurrence rate of stroke following PFO closure, though older individuals exhibited a slightly higher recurrence rate (33).

A meta-analysis revealed a 41% decrease in recurrent stroke rates following closure, particularly in patients with high-risk PFOs (34). The CLOSE trial demonstrated that PFO closure combined with antiplatelet therapy was superior to antiplatelet therapy alone in preventing stroke recurrence aneurysm [with a hazard ratio of 0.03 (95% CI, 0 to 0.26; *p* < 0.001)]. This benefit was most pronounced in patients with high-risk PFO features such as large shunts or atrial septal. The secondary composite outcome of stroke, transient ischemic attack (TIA), or systemic embolism was significantly lower in the PFO closure group compared to the antiplatelet-only group (3.4% vs. 8.9%; hazard ratio = 0.39, 95% CI = 0.16–0.82, *p* = 0.01) (35).

However, complications such as atrial fibrillation were reported in 4.6% of cases following PFO closure, a rate significantly higher than in patients managed with antiplatelet therapy alone (*p* = 0.02); the impact of AF secondary to PFO closure on stroke risk remains unclear (36). In another study the incidence of atrial fibrillation (AF) after PFO closure was reported at <5%, peaking around day 14 post-closure and declining after day 45 (37). The pathophysiological mechanisms behind post-PFO closure AF are not well understood but may include local irritation, device-related interference, tissue stretch, and nickel hypersensitivity. Management strategies focus on rhythm control, with flecainide showing promise, and anticoagulation tailored to individual risk profiles. Post-closure AF is generally benign and resolves within 45 days, minimizing thromboembolic risks (37). Preexisting AF may also be uncovered through intensive diagnostic strategies (7).

The CLOSURE 1 trial, which evaluated the efficacy of the STARFlex septal closure system, found no significant advantage of PFO closure over medical therapy (38). Furthermore the periprocedural major vascular complications occurred in 3.2% of patients in the closure group. The Kaplan–Meier estimates of 2-year rates of stroke were 2.9% in the closure group and 3.1% in the medical-therapy group, and respective rates of 3.1 and 4.1% for TIA. The key findings of the study were that there was no significant difference between the two treatment groups in the rate of recurrent stroke or TIA (38). Conversely, the REDUCE trial supported the efficacy of PFO closure, reporting a significantly lower risk of recurrent stroke compared to antiplatelet-only therapy (39). The study found that clinical ischemic stroke occurred in 1.4% of patients in the PFO closure group and in 5.4% of patients in the antiplatelet-only group. Also the incidence of new brain infarctions was significantly lower in the PFO closure group than in the antiplatelet-only group [18 patients (4.7%) vs. 19 patients (10.7%)] (relative risk = 0.44, 95%, CI = 0.24 to 0.81, *p* = 0.02), but the incidence of silent brain infarction did not differ significantly between the study groups (*p* = 0.75). Atrial fibrillation or flutter occurred in significantly more patients in the PFO closure group than in the antiplatelet-only group (6.6% vs. 0.4%, *p* < 0.001); 83% of the cases of atrial fibrillation or flutter were detected within 45 days after the procedure, with 59% of them being resolved within 2 weeks after onset (39). Recurrent stroke rates after PFO closure was slightly higher in patients aged 18–45 compared to those aged 46–59 (1.5% vs. 1.3%, respectively). Contrary to that, in a meta-analysis by Xu et al. (40) younger patients had fewer outcomes of recurrent neurological episodes after PFO closure. Noteworthy is that in diagnosed AF concomitant with PFO, PFO closure is not the best option since there is no clear way to rule out the PFO as being merely an incidental factor (36).

### Biomarkers

Biomarkers hold significant promise as tools for evaluating PFO-related strokes. Evidence indicates that total homocysteine (tHcy) levels are markedly reduced following PFO closure, particularly in cases of complete closure (41). In contrast, medical therapy alone does not appear to influence tHcy levels. Advanced analytical techniques such as metabolite profiling, orthogonal partial least squares discriminant analysis (OPLS-DA), and two-way repeated-measures ANOVA have been employed to identify metabolites associated with PFO closure. Furthermore, mixed-effects model repeated measures analysis was used to assess the impact of residual shunting and PFO treatment on tHcy levels. The findings revealed that PFO closure significantly reduces peripheral blood tHcy levels, while residual shunting is independently associated with elevated tHcy levels (41).

Additional findings suggest improvements in dynamic cerebral autoregulation and reductions in platelet-derived growth factor-BB, a marker often elevated in PFO populations (42, 44). Concept of stroke-related biomarkers provide avenues for further research.

## Multidisciplinary approach

Effective management of cryptogenic stroke with PFO requires a collaborative approach. Neurologists are essential for assessing stroke symptoms, interpreting neuroimaging, and ruling out other etiologies. Collaboration with cardiologists and radiologists is crucial for making appropriate management decisions. Below is a flowchart summarizing the decision-making process for cryptogenic stroke with suspected PFO:

**Table:** 

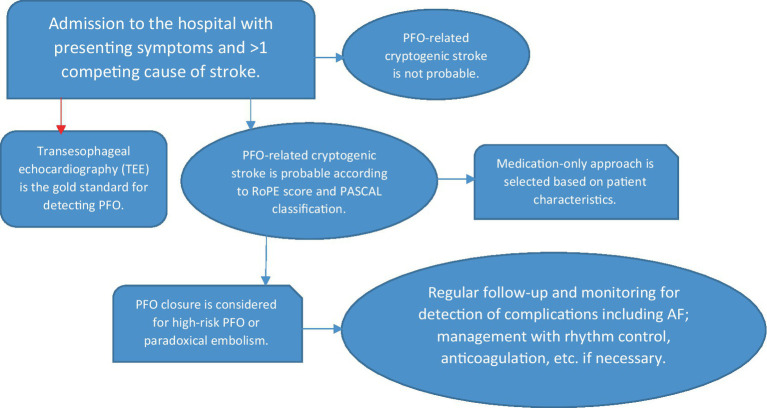

## Discussion

While PFO is strongly associated with cryptogenic stroke, but its presence does not confirm causality, since it may also be an incidental finding. Biomarkers provide additional data about stroke risk and the proper intervention. However, challenges such as accurately selecting patients for PFO closure and evaluating the true likelihood of PFO being the causative factor in cryptogenic stroke remain areas of uncertainty. Multidisciplinary collaboration and ongoing research, particularly longitudinal studies and randomized controlled trials, are essential to guide us in the endeavor of understanding the mechanisms linking PFO and cryptogenic stroke and addressing the enigmatic nature of these events.
